# Supplementary material for: Network Pharmacology Analysis and Experimental Validation of Kaempferol in the Treatment of Ischemic Stroke by Inhibiting Apoptosis and Regulating Neuroinflammation Involving Neutrophils
Source: Int J Mol Sci. 2022 Oct 21;23(20):12694. doi: 10.3390/ijms232012694 (PMC9604352; doi:10.3390/ijms232012694)
Supplement: Supplementary file 1 [file ijms-23-12694-s001.zip › Supplementary Figures.pdf]

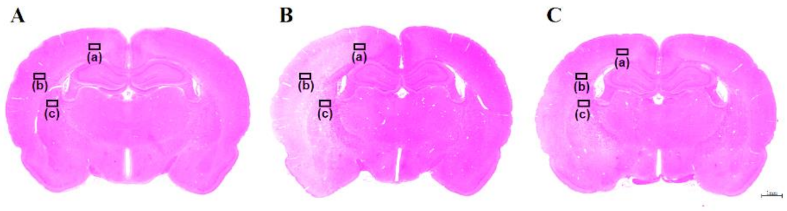

Figure S1 Representative images of the location of (a) penumbra, (b) ischemic core and (c) striatum in ischemic brain of (A) Sham group, (B) I/R group and (C) I/R + KAE-100 group.

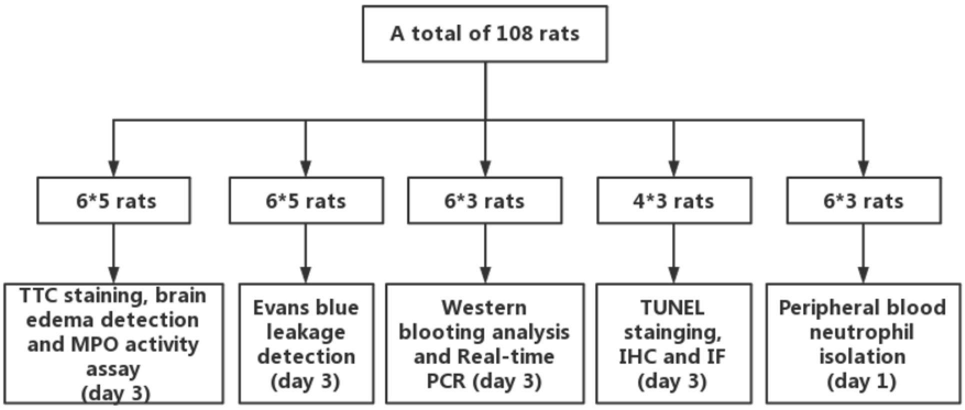

Figure S2 The exact number of rats used in each experiment.

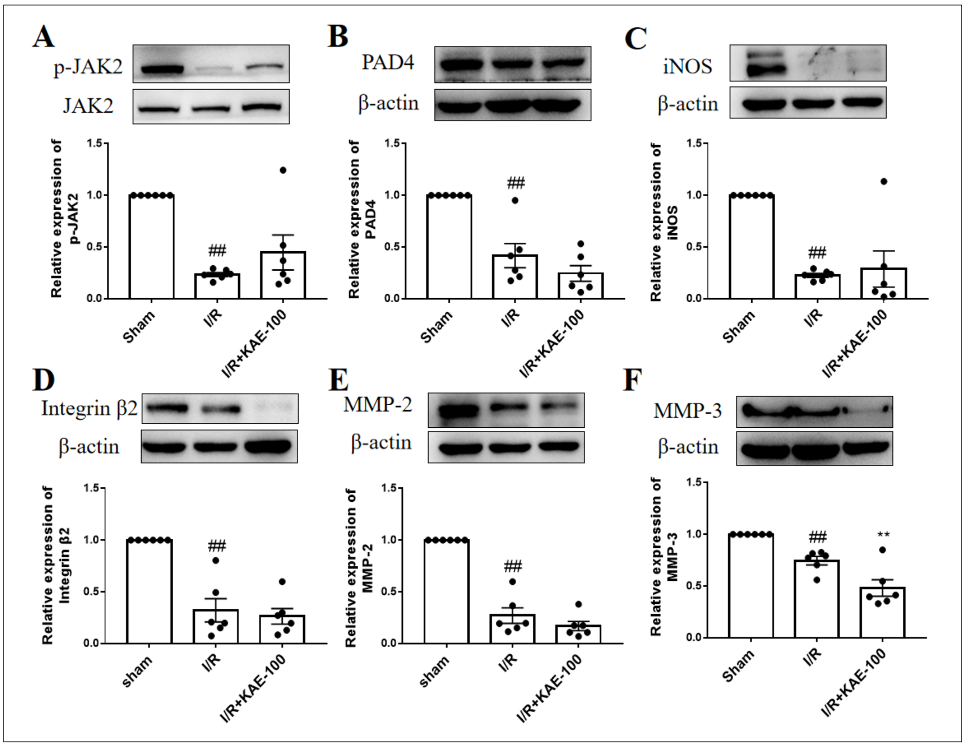

Figure S3 The expression level of p-JAK2 (A), PAD4 (B), iNOS (C), Integrin β2 (D), MMP-2 (E) and MMP-3 (F) in ischemic cortex of rats on day 3 after I/R. Values are expressed as mean ± SEM. ## p <0.01 vs. Sham group; ** p <0.01 vs. I/R group.
